# Genetic Diversity Analysis and Core Germplasm Construction of *Rubus chingii* Hu

**DOI:** 10.3390/plants13050618

**Published:** 2024-02-23

**Authors:** Ziwei Zhou, Fen Liu, Yanqin Xu, Weiming Hu

**Affiliations:** 1 College of Pharmacy, Jiangxi University of Chinese Medicine, Nanchang 330004, China; zhouziwei@jxutcm.edu.cn; 2 Lushan Botanical Garden, Jiangxi Province and Chinese Academy of Sciences, Jiujiang 332900, China; liuf@lsbg.cn

**Keywords:** *Rubus chingii* Hu, SNP, genetic diversity, population structure, core collection

## Abstract

*Rubus chingii* Hu is the only species that is used for both edible and medicinal purposes among the 194 species of the genus *Rubus* in China. It is well known for its sweet and sour fresh fruits that are rich in vitamins and for its dried immature fruits that are used to treat kidney-related ailments. This study aims to evaluate genetic diversity and population structure and build a core germplasm repository of 132 *R. chingii* accessions from the provinces of Jiangxi and Fujian, using Hyper-seq-derived single-nucleotide polymorphism (SNP) markers. This is the first genetic study of *R. chingii* based on SNP molecular markers, and a total of 1,303,850 SNPs and 433,159 insertions/deletions (InDels) were identified. Low values for observed heterozygosity, nucleotide diversity (Pi) and fixation indexes (Fis) indicated low genetic diversity within populations, and an analysis of molecular variance (AMOVA) showed that 37.4% and 62.6% of the variations were found between populations and within samples, respectively. Four main clusters were identified by means of neighbor-joining (NJ) trees, the ADMIXTURE program and principal component analysis (PCA). Based on the genetic diversity, we finally constructed 38 representative core collections, representing 50% of the total core germplasm samples and 95.3% of the genotypes. In summary, the results of our study can provide valuable information on the genetic structure of *R. chingii* germplasm resources, which is helpful for further explorations of potential high-quality genes and for formulating future breeding and conservation strategies.

## 1. Introduction

*Rubus chingii* Hu (named Zhangye-Fupenzi in China) is a diploid species (2n = 2x = 14) and a perennial rattan shrub of the *Rubus* genus of the Rosaceae family, distributed in southern Anhui, Zhejiang, northern Fujian and northeastern Jiangxi in China [1,2,3]. Of the 194 Chinese *Rubus* species, *R. chingii* fruit was the only one included in the *Pharmacopoeia of the People’s Republic of China 2020* and the homologous catalog of medicine and food by the National Health Commission of China in 2015; it is also listed as “the third-generation fruit” in the “new fruit trees in the 21st century” [4,5,6,7,8]. The dried and unripe fruit of *R. chingii* harvested at the green-to-yellow stage has been the basic source of the traditional Chinese medicine (TMC) known as Fupenzi for more than 1500 years; it has mild medicinal properties and is beneficial to the kidney, strengthening the essence of and shrinking urine, nourishing the liver and improving eyesight. In addition, it is rich in health-promoting components such as terpenoids, flavonoids, alkaloids and phenolic acids [9]. Modern pharmacology shows that Fupenzi has antioxidant, anti-inflammatory, anti-tumor and other effects [5,10,11,12]. The ripe fruit has a high sugar–acid ratio and a good taste when eaten fresh, and it contains rich trace elements. It has been used in food, drink, health care and other industries, as well as in jam and raspberry wine [13,14]. Previous research has shown that variations in the fruit at different phases of growth arise from the coordinated accumulation of flavonoid and phenolic acid syntheses at various stages and their subsequent conversion into derivatives [15].

Zhejiang Province and the northeastern region of Jiangxi Province are the primary production areas for *R. chingii* [16]. Planting areas in the provinces of Zhejiang and Anhui have grown significantly in recent years, creating a distinct scale for the combined agriculture and tourism industries; with the ongoing rise in market demand, its commercial benefits are outstanding, and its future potential is vast [1,17]. Its new plant variety certificate was achieved by domesticating *R. chingii* natural resources into cultivars, and a summary of its introduction, domestication, propagation and cultivation procedures has been provided [17,18]. These techniques primarily involve the analysis of branching, leafing, fruiting traits and medicinal components, as well as the screening of good plants with the potential for individual application and popularization [19,20,21]. However, narrow genetic resources and limited breeding strategies superadd the interaction of genotypes and the environment lowers the efficiency of choosing excellent material [22,23]. This is coupled with the fact that *R. chingii* mainly propagates root tillering seedlings [24], resulting in variety degradation and lower yields [25]. Utilizing molecular markers to encourage *R. chingii* assistant breeding methods can help overcome this obstacle and hasten improvement.

Plants in the genus *Rubus* are primarily classified and identified based on differences in phenotypes and chromosomal composition [26]. An effective method for analyzing the genetic diversity of germplasm resources involves molecular markers [27]. Numerous researchers have previously studied the relationship and genetic diversity of *R. chingii* on the bases of the internal transcribed spacer (ITS), random amplified polymorphic DNA (RAPD) and inter-simple sequence repeat (ISSR), thus demonstrating the high feasibility and effectiveness of using molecular marker methods for germplasm resources research [28,29,30,31]. In addition, the chromosome-scale reference genome of *R. chingii* was sequenced and assembled by Wang et al., yielding 231.21 Mb of sequence data; moreover, 1,817,604 such SSR sequences were found in the genome [32]. Jiang et al. used transcriptome data to mine SSR sequences and annotate functional genes in the SSR-containing region [33]. More improved knowledge is needed of *R. chingii*’s transcriptome and genomic coding sequences to facilitate the use of more straightforward and effective single-nucleotide polymorphisms (SNPs) and insertion/deletion (InDel) markers for *R. chingii* genetic diversity and molecular breeding.

The distribution of *R. chingii* resources is widely dispersed, and there are exceedingly low numbers in the wild. This, combined with harmful human exploitation, makes the damage extremely severe [34]. Moreover, there is a paucity of the literature about the evaluation and collection of germplasm resources for *R. chingii*. The majority of research subjects come from the provinces of Anhui and Zhejiang, while hardly any studies have been conducted on those originating from the Jiangxi and Fujian provinces [20,30,31,33,35]. It takes a lot of time and effort to collect and preserve as many genetic resources as possible, despite the fact that germplasm conservation is crucial for biodiversity and plant breeding [36]. Core collection can represent the genetic diversity of all of the species’ resources to the greatest extent, with the least amount of genetic duplication, which can enhance the management and utilization efficiency of germplasm resources; thus, the core collection has become the focus of plant germplasm resources research, both domestically and internationally [37,38,39]. For the purpose of the conservation and sustainable use of *R. chingii* resources, it is crucial to investigate the genetic diversity and distribution of *R. chingii* in the field. In addition to revealing genetic traits, analyzing the genetic diversity, phylogenetic relationships and population structure of *R. chingii* germplasms can serve as a foundation for germplasm identification, resource conservation and utilization, and effective breeding. However, there are few studies on the genetic diversity and population structure of *R. chingii* based on DNA molecular markers. This, coupled with its unclear domestication history and extremely complicated genetic background, has created a bottleneck for the application of effective breeding strategies.

Advances in next-generation sequencing technology have made whole-genome sequencing more efficient and cost-effective than ever before, and they offer the opportunity to find a large number of DNA polymorphisms in the genome, such as SNPs and InDels [40]. SNPs are the most common variants in the genome of any organism [41], and InDels have become an increasingly important source of genetic variation [42]. Despite the continuous development of genotyping techniques for SNPs, InDel polymorphisms are easily genotyped by fragment-length polymorphisms and are of practical value for laboratories that do not have the infrastructure to perform SNP genotyping [43]. They are best suited for genetic evaluation and strategies for selective breeding using molecular genetics.

Hyper-seq is an extremely low-cost, efficient, flexible and high-throughput DNA sequencing library preparation and genotyping method that was developed by Xia’s team at Hainan University [44]. This technique mainly consists of PCR amplification to construct the library and gel electrophoresis to preliminarily detect the quality of the library. Then, the mixed glue is recovered, and the quality of the library is controlled again by means of Nanodrop and gel electrophoresis; the second-generation Illumina NovaSeq 6000 platform is used for high-throughput sequencing and for several key steps, such as quality control and filtering of the original data generated by sequencing. This technology has wide applicability and scalability, as well as a certain gene region enrichment effect. Utilizing various Hyper-seq primers, the label density can be readily modified to suit the requirements of various species and projects. Additionally, special PCR techniques eliminate the need for additional enzyme digestion and joint procedures, which can realize the simultaneous construction of a large number of samples, produce massive genotype big data, meet the needs of large-scale typing sequencing of different species at low cost and accelerate efficient big data breeding and population research. Wang et al. used Hyper-seq technology to conduct genome-wide association analysis (GWAS) on 150 tetraploid potatoes, and they discovered candidate genes that may be closely related to the formation and regulation of the flesh colors of potato tubers [45]. Fu et al. combined Hyper-seq sequencing of 241 *Canna edulis* populations to identify key genes related to leaf color and morphology and completed the classification of *C. edulis* populations [46]. Ding et al. constructed a sequencing library of 137 *Areca catechu* DNA samples using the Hyper-seq method and mined 86 candidate genes related to *Areca catechu* fruit shape traits [47].

In the current study, 132 individuals from 11 wild populations of *R. chingii* in Jiangxi and Fujian provinces were subjected to simplified genome sequencing conducted with Hyper-seq technology. The primary objectives of this study were to (1) evaluate the genetic diversity and population structure of *R. chingii* accessions and (2) develop a core germplasm set, conserving diversity for improvement and breeding programs. This is the first genetic study on *R. chingii* that uses very accurate SNP molecular markers, and it offers a theoretical foundation for the comprehension, preservation and sustainable use of wild *R. chingii* resources in Jiangxi and Fujian provinces. Moreover, these two significant *R. chingii*-producing regions have an abundance of wild resources, which are crucial for enhancing the species’ excellent germplasm resources and expanding their gene pool.

## 2. Results

### 2.1. Genome Re-Sequencing and Variant Identification

The re-sequencing of 132 accessions of *R. chingii* was performed with the Illumina sequencing platform. The total depth of sequencing was 309.92×, and approximately 70.17 G of sequencing data was generated. We extracted 493 million clean reads, with an average of 3.7 million reads per individual, by filtering low-quality reads and reads less than 15 bp in length. The mean value of Q30 was 91%, and the GC content was between 37.48% and 44.76%, which indicates high library quality and accurate and reliable sequencing results that can be used for subsequent SNP marker mining (Supplementary Table S1). Clean reads of each accession were mapped onto the *R. chingii* reference genome using a BWA aligner. The percentage of reads mapped onto the reference genome varied from 70.26% to 97.27% (Supplementary Table S2).

**Figure 1:**
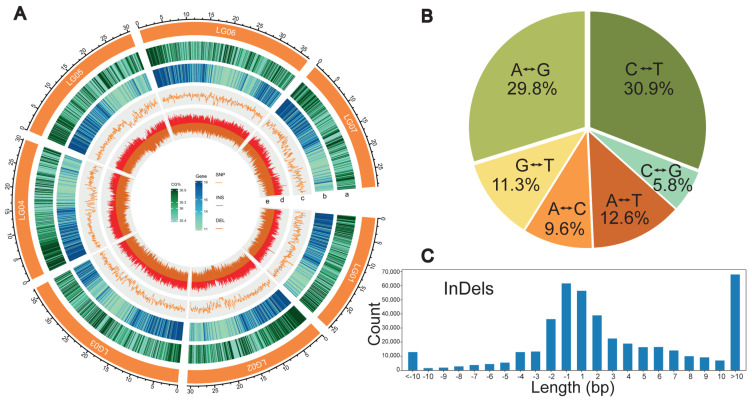
SNPs and InDels screening and in silico simulation. (**A**) Genome-wide variation distribution. Tracks toward the center: a, CG content (%); b, number of genes; c, number of SNPs; d, number of insertions; e, number of deletions. (**B**) Proportion of six variant types of SNPs in the whole population. (**C**) Distribution of InDel lengths.

A total of 1,303,850 SNPs and 433,159 InDels were identified, located on 7 chromosomes and 35 scaffolds (Supplementary Figures S1–S3); they were considered as a candidate pool for further selection and were evenly distributed across the *R. chingii* genome (Figure 1A). The number of alleles (Na) varied from two to seven (Supplementary Table S3), while the length distribution of InDels was within 10 bp, and 1 to 2 bp were the two most abundant types, accounting for 45% (Figure 1C). The overall mean SNP and InDel densities of the chromosomes were 3.5 SNPs/kb and 1.4 InDels/kb. Chromosome 6 had the highest frequency of SNPs (6.782 SNPs/kb) and InDels (2.655 InDels/kb), while chromosome 5 had the lowest (Supplementary Table S4).

Transitions (Ts) SNPs (A/G or C/T) were more abundant than transversions (Tv) SNPs (A/C, A/T, C/G or G/T), with a Ts/Tv ratio of 1.54. (Supplementary Table S5). The single base variation showed that C/T was the dominant conversion type, accounting for 30.9%, while C/G conversion represented only 5.8% (Figure 1B).

The identified SNPs and InDels were annotated to identify the genes disrupted by the variants and to assess the effect of the mutation on individuals. The results showed that nonsynonymous single-nucleotide variants (SNV) accounted for up to 41.5% of SNPs, synonymous SNV accounted for 21.8%, and frameshift deletions and frameshift insertions accounted for 6.7% and 10%, respectively. InDels identified the largest number of mutations in the intergenic part of the genome, accounting for 33.9%, followed by intronic, accounting for 24.1%, and exonic, accounting for 19.2% (Supplementary Table S6).

### 2.2. Genetic Diversity

The main allele frequency (MAF), expected/observed heterozygosity, expected/observed homozygosity, nucleotide diversity (Pi) and fixation index (Fis) and other parameters were used to assess genetic diversity. The number of polymorphic sites and mean number of individuals per locus values (Num Indv) of these SNPs ranged from 21,693 to 250,386 and from 1 to 11.4, respectively. The percentages of locations with polymorphisms ranged from 26,621 to 231,860 and from 7.34% to 25%, with a mean value of 15.76%. MAF had an average of 0.957, ranging from 0.932 to 0.972. The expected and observed heterozygosities had mean values of 0.053 and 0.044, ranging from 0.034 to 0.090 and from 0.033 to 0.073, respectively. Meanwhile, the expected and observed homozygosities had average values of 0.947 and 0.956, ranging from 0.910 to 0.965 and from 0.926 to 0.969, respectively. The Pi and Fis values ranged from 0.047 to 0.115 and from 0.012 to 0.186, respectively, with mean values of 0.0738 and 0.061, respectively, as shown in Table 1.

The results of inter-populational molecular AMOVA showed that most of the variance occurred among individuals, accounting for 62.6% of the total variation, and a further 37.4% of the total variation was attributed to inter-populational differences. The genetic differentiation coefficient (Phi) was 0.37 (*p* < 0.05) (Table 2).

The fixation index (Fst) value of the 11 populations ranged from 0.13 to 0.46, suggesting high genetic differentiation among populations (Supplementary Table S7). In addition, the lowest Fst value was 0.13 between populations 30 and 14; the highest Fst value was 0.46 between populations 18 and 17.

### 2.3. Population Structure

**Figure 2:**
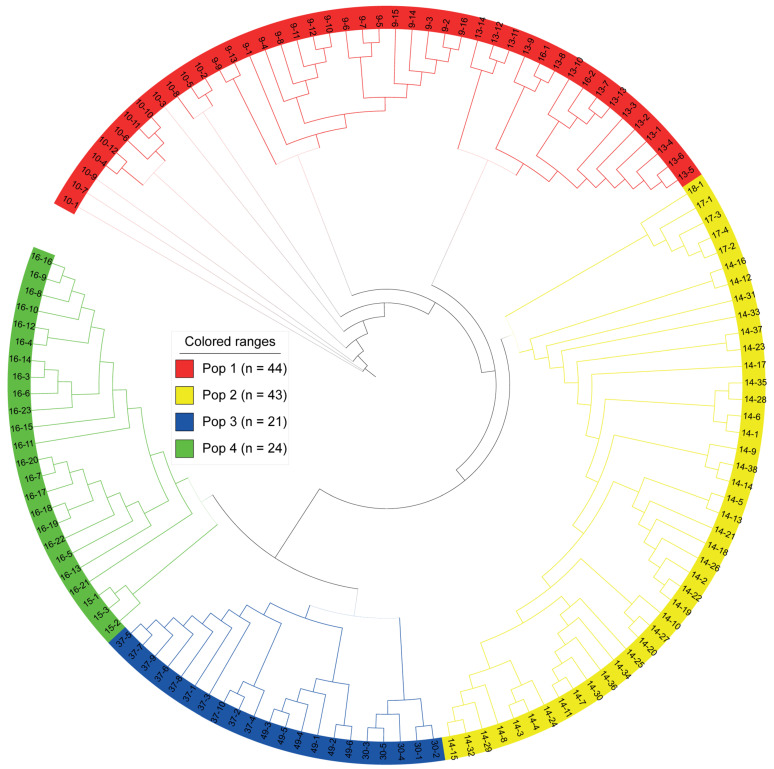
Neighbor-joining phylogenetic tree of 132 *R. chingii* accessions using SNP data. Different inferred populations are distinguished by different colors.

To infer relationships among the 132 accessions of *R. chingii*, a neighbor-joining (NJ) tree was constructed. PLINK software (version 3.696) was utilized with VCF files to calculate squared genetic distances between individuals based on SNP data. Four subgroups were generated from this NJ tree. Pop1 (red in Figure 2) included all the resources in populations 9, 10 and 13 and two accessions in population 16, while the genetic structure of populations 14, 17 and 18 showed a closer relationship to Pop2 (yellow in Figure 2). Pop3 (blue in Figure 2) consisted of 21 accessions from three populations: 30; 37; and 49. Pop4 (green in Figure 2) comprised 24 accessions, population 15 and the remaining 21 accessions of population 16. In general, except for a small internal division from population 16, individuals from every other population clustered on the same genetic branch.

**Figure 3:**
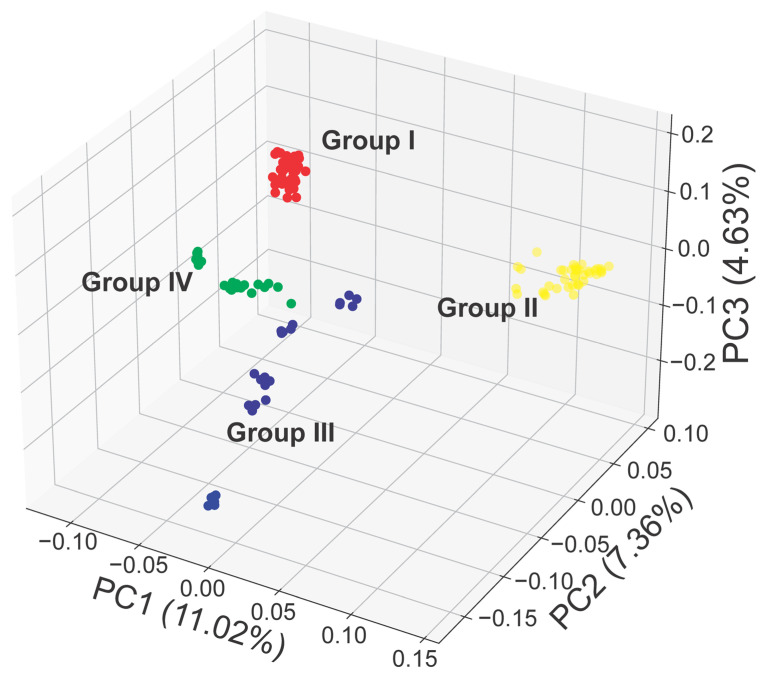
Principal component analysis (PCA) on the 132 *R. chingii* accessions.

Meanwhile, the principal component analysis (PCA) obtained using the SNP markers generated in this study provides useful information on the relationships among *R. chingii* accessions and is generally consistent with the results observed in the NJ tree (Figure 3).

**Figure 4:**
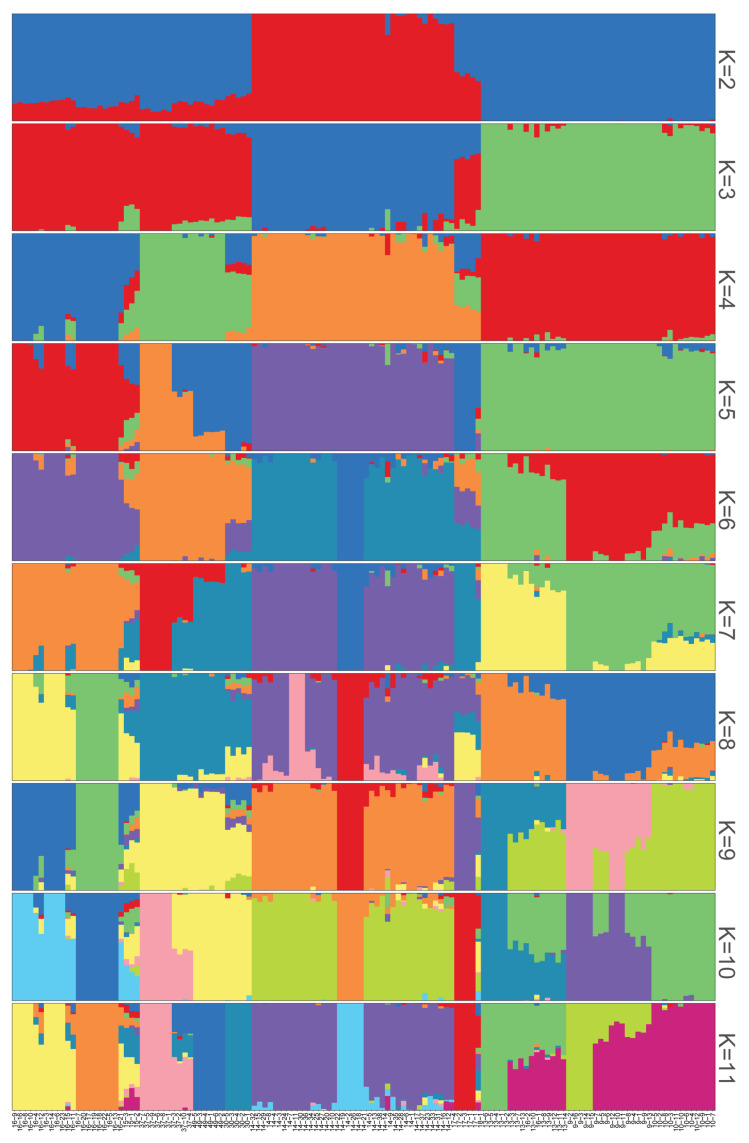
Population structure of 132 *R. chingii* accessions at K = 2–11. The square columns represent samples; the colors correspond to the origins of the ancestors, and the proportions of the colors represent the proportions of the ancestries in the sample.

In the ADMIXTURE analysis, the most probable K value varied from 2 to 11, with K = 4 having the lowest cross-validation error (Figure 4, Supplementary Figure S4). Thus, K = 4 was considered the optimal number of subpopulations, indicated as clusters I–IV to describe the genetic structure. The NJ tree results and PCA analysis supported the ADMIXTURE study.

### 2.4. Core Germplasm

**Figure 5:**
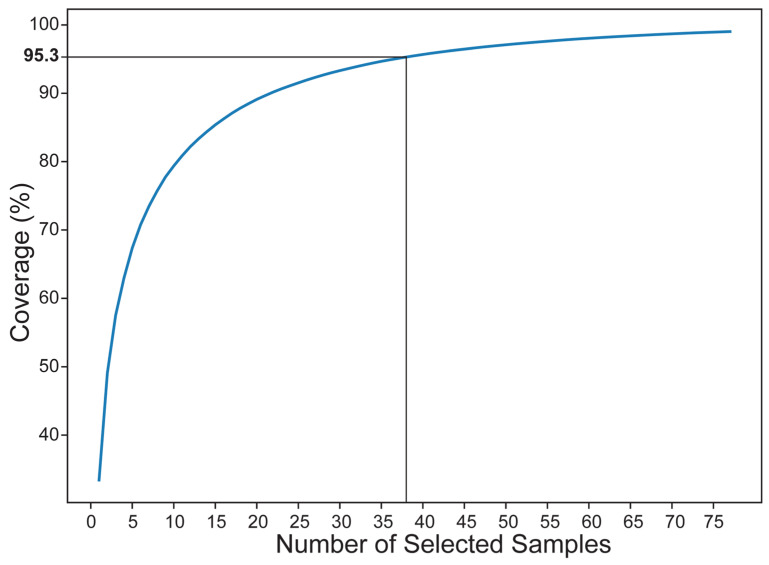
Genotype coverage trend map. When the sample size is 50, the genotype coverage reaches 95.3%.

In this research, the core germplasms of *R. chingii* were constructed from 132 wild accessions using a combination of Hyper-seq technology and Genocore [48]. According to the NJ tree, there are 21 accessions in Pop1, accounting for 27.3%; 28 accessions in Pop2, accounting for 36.4%; only 14 accessions in Pop3, accounting for 18.2%; and 14 accessions in Pop4, accounting for 18.2%. Furthermore, 38 accessions of *R. chingii* samples were chosen from the core set if the sample was 50% (Table 3); the genotype coverage is 95.3% (Figure 5, Supplementary Table S8), and Pop2 contains 14 accessions, reaching the maximum of 36.8%. At this time, the noncore germplasm contains 27 strains. The percentage of polymorphic sites of the core germplasm population is 91.5%; the effective allele number is 1.4293, and the Shannon’s information is 0.6192. Nei’s gene diversity is 0.6341, higher than that in the noncore germplasm set. The average observed heterozygosity of the core germplasm population is 0.1833, which is slightly lower than that of the noncore germplasm sets.

## 3. Discussion

### 3.1. SNPs and InDel Markers

On the basis of the published *R. chingii* genome, it becomes easy and quick to mine the genomic SNP and InDel markers by means of re-sequencing and bioinformatics. Single-nucleotide polymorphisms refer to single base differences that exist in the genomes of different individuals of a species, and they are also a rich form of genetic variation within individuals of a species that can occur at different frequencies throughout the genome [49,50]. In this study, genotype DNA libraries for *R. chingii* were created and genotyped using Hyper-seq technology. Then, the SNP markers were utilized to examine the genetic diversity and population structure. Previous studies carried out *R. chingii* ISSR marker studies and transcriptome SSR mining and analysis [31,33], whereas SNPs are being used here for genotyping for the first time.

The alignment efficiency between the results of this study and the reference genome is from 70.26% to 97.27%, which reveals that there are differences in the whole-genome sequence of the materials studied. The average ratio of Ts/Tv is 1.54; similar rates exist in other plants, like sweet cherry and sorghum [51,52]. Furthermore, some reports suggest that high Ts/Tv ratios indicate low levels of genetic differentiation in genomic comparisons [53]. Nevertheless, transversion is more likely to change the amino acid sequence of proteins, suggesting that transversion has a greater influence on the regulation of DNA, and the local deviation of the Ts/Tv ratio can also reflect the evolutionary selection of genes [54,55]. Insertions or deletion variants of 1–2 bp are the most common type, and the size of InDels is negatively correlated with their abundance, which has also been found in previous studies with different crops [53,56,57]. In addition, the distributions of SNPs and InDels vary with the type of sequence region, but the distributions are not uniform; the distribution densities differ on various chromosomes, and the frequency of polymorphism in intergenic regions is relatively higher than that in gene regions [58]. Moreover, variations located within or close to coding sequences should always raise greater concerns due to the increased likelihood that they will be connected to a particular biological function [59]. In this study, the numbers of SNPs and InDels found on chromosome 6 are the largest, while their distribution on chromosome 5 is the smallest, which was mutually verified with the conclusion that InDels events were positively correlated with single-nucleotide changes [60]; this also indicates that the diversity of chromosome 5 is low. The largest number of InDel variants were identified in the intergenic part of the genome, but no variants were detected in the 3′ UTR and 5′ UTR parts, similar to the findings of the Chinese cabbage study [61]. The newly identified SNPs and InDel markers can provide abundant data information for genetic and functional genomics studies of *R. chingii,* quickly identify dominant populations, provide a deeper understanding of the genomic diversity and population structure of germplasm and establish a foundation for the continued breeding of superior species.

### 3.2. Genetic Diversity Analysis

Genetic diversity is critical for a healthy population because it represents different alleles that can lead to resistance to pests, diseases or other stressful conditions; it is essential to retain sufficient genetic diversity for current and future plant breeding programs [62]. However, the current rate of species extinction is rapidly approaching an unprecedented level, with conservative estimates of genetic diversity within wild populations declining by 5.4–6.5% since the Industrial Revolution, and the rate of biodiversity loss does not appear to be slowing down. A better understanding of the genetic diversity characteristics, population structure and ecological relationships of wild resources is necessary to develop and implement effective genetic conservation strategies [63,64,65]. From a molecular level, this study analyzed genetic diversity in wild *R. chingii* species in order to further provide a foundation for genetic resource protection and a basis for the sustainable utilization of resources.

Regarding the observed heterozygosity, all of the observed heterozygous population SNPs loci were lower than expected, and *R. chingii* (0.044) had a much lower observed heterozygosity than *Prunus persica* (0.444) [66]. This indicates a clear lack of heterozygosity and low genetic diversity that may herald a potentially depressed breeding problem [66,67]. The high MAF (0.95) and Fis > 0 confirmed that there is less observed heterozygosity than expected, which also indicates that the population has a low outcrossing rate and low genetic variation, which may be related to the characteristics of root tiller reproduction [68,69].

The major drivers of genetic diversity loss include climate change, habitat fragmentation, overcollection and population size reduction [70]. Considering that genetic variations within and between populations do not depend on sexual and/or asexual reproduction, this means that sexual plants are as genetically diverse as asexual plants [71,72]. Overexploitation of nature often results in habitat loss for wild resources, while habitat fragmentation leads to smaller population sizes, which will also endanger the long-term survival of *R. chingii* through asexual reproduction and genetic drift [66]. Therefore, it seems that poor habitat conditions, low distribution density and severe human intervention are the main reasons for the reduction in genetic diversity [73]. However, the small number of materials in some regions may not be a true reflection of the low diversity level, and further studies with larger samples are needed.

### 3.3. Population Structure

To effectively utilize germplasm resources and safeguard variety rights, access to genetic relationships and population structure at the genomic level is required [74]. There is some evidence of significant genetic differentiation in wild *R. chingii* populations in the current investigation. Firstly, the amount of variance between populations is further quantified by the interpopulation fixed index (Fst) [69]. Strong genetic divergence between populations is indicated by an average Fst value of 0.253 [67]. The highest Fst values were found between populations 17 and 18, indicating the highest degree of genetic differentiation between them. Surprisingly, the Fst values of the two largest sampled populations, 14 and 16, are both lower than 0.25, indicating a modest degree of differentiation between these two populations. Conversely, population 18, with the smallest sample size, had a high level of differentiation, shown by its Fst score. The broadest range of alleles may be covered by the large sample size, and since there is more genetic overlap with other groups, there may be less genetic differentiation. Secondly, the results of the AMOVA study supported earlier findings that most woody species change more between individuals than across populations, with the majority of variations occurring within samples [75,76]. The Phi among the samples reached a significant level of 0.37 (*p* < 0.05), and there was also a high level of genetic differentiation demonstrated between monoculture materials [77].

The results of the NJ tree, ADMIXTURE structural and PCA analyses all divided 132 wild *R. chingii* resources into four subgroups. Regular patterns in the classification of germplasm resources are mostly influenced by known material lineages, geographical origins and dissemination patterns [51]. Pop1 is mainly located in the northeast of Jiangxi Province, while Pop3 distribution is concentrated in Jiangxi’s center; the main distribution of Pop4 is in Fujian, which borders Jiangxi; and Pop2 is dispersed across both Jiangxi and Fujian provinces, roughly 400 km apart, which is not consistent with the criteria of geographical origin of the first three subgroups. In addition, population 16 was classified into two subgroups. Given that *R. chingii*’s natural resources are primarily found in places where there has been significant human disturbance, like hillsides and roadside areas, and its fruits are favored by birds and animals, its seeds may also spread with their range. Thus, the first possibility that comes to mind is that transmission mediated by humans or animals may be involved [2,78,79]. *R. chingii* germplasm resources were not strictly categorized based on the established population, and a subpopulation can be further divided into distinct groups, each exhibiting some degree of confounding, which indicates a varied genetic makeup within each of these subgroups [68,69].

Only population 14 arose when K = 2, according to the structural analysis, suggesting that this population’s differentiation period may have occurred earlier in the evolutionary process. When K = 3, Pop3 and Pop4 were formed, which were distributed in central Jiangxi Province and Fujian Province but not separated and had been isolated from Pop1 located in northeastern Jiangxi Province, indicating that *R. chingii* was likely to migrate from northeastern Jiangxi Province to central Jiangxi Province and Fujian Province, where it would likely spread quickly [80,81]. Although cross-cutting between materials and environmental factors may allow the populations of different origins to belong to the same subgroup, most species of the same origin with similar genetic background information may be categorized accurately. The ADMIXTURE analysis results show that for K = 4, populations 15, 30, 17 and 18 have a high proportion of color mixing, making it difficult to distinguish between them based on gene pools of different colors.

### 3.4. Core Germplasm Repository Building

This study demonstrates that heterozygote deficits are present in all groups of wild *R. chingii* populations, and that these populations maintain high levels of genetic differentiation and low levels of genetic variation. Therefore, effective *R. chingii* conservation strategies should be proposed based on the population’s genetic diversity information. Maintaining the greatest amount of genetic variation should be the primary objective of any program aimed at conserving plant genetic resources [82]. The quantity of the *R. chingii* core germplasm building group is far lower than that of the total sample, but its genetic diversity index, such as observed heterozygosity, is higher than that of each sample population. This is because the core germplasm is protected with a minimum number of genetically similar materials, which increases genetic diversity [83]. Since different needs and crops require varying sample percentages, there is no perfect ratio or set size for all core germplasm sets. The genotype coverage trends indicate that when the genotype coverage approaches 95%, the percentage of matched core germplasm grows marginally with sample size, and at this point, it is approximately 50%. Consequently, 50% of core germplasm was deemed to be the best core germplasm in this investigation, and at this point in time, the observed heterozygosity was 0.1833, which was higher than that of any population. Nei’s gene diversity (0.6341) and Shannon’s information (0.6192) indicated a high genetic variation level. Both in situ and ex situ conservation are required since *R. chingii* is a widely distributed species with a large range, and no single conservation strategy is optimal [62]. Core germplasm nurseries provide valuable information for germplasm conservation, which can be followed by joint phenotypic trait analysis used to develop genetic populations to scan target loci and genes and select parental material to improve breeding.

## 4. Conclusions

Research on the genetic diversity and population structure in populations of wild resources is crucial for comprehending the status of these resources, as well as for discovering beneficial genes and generating new cultivars. It provides a strong scientific basis for understanding how various species adapt to their surroundings and for creating workable plans for the conservation and utilization of genetic resources. Following filtering, 1,303,850 SNP polymorphic loci and 433,159 InDel polymorphic loci were discovered using Hyper-seq on *R. chingii* wild resources. Based on the genetic diversity and AMOVA studies, *R. chingii* in Jiangxi and Fujian provinces has maintained a low level of genetic variation, suggesting that its genetic integrity may be at risk. Meanwhile, its high degree of population differentiation suggests that material should be collected from a range of populations in order to maximize the genetic variance of the germplasm. Four subgroups can be formed by combining a NJ tree, ADMIXTURE analysis and PCA, and their genetic distances are the primary factors that determine the priority of the major categorization. According to the genotype coverage trends, when 38 strains were sampled, the core germplasm was built, and the genotype coverage was 95.3%. The majority of genetic diversity was preserved using a modest amount of germplasm resources. In addition, to preserve the integrity of the habitat and lessen the logging of wild resources, a combination of in situ and relocated conservation techniques should be used. In order to apply molecular breeding, consideration should also be given to the discovery of alleles for important features in the natural resources of the populations. The genetic diversity and population structural data from this research can serve as a foundation for *R. chingii* conservation, management and further utilization.

## 5. Materials and Methods

### 5.1. Experimental Materials

**Figure 6:**
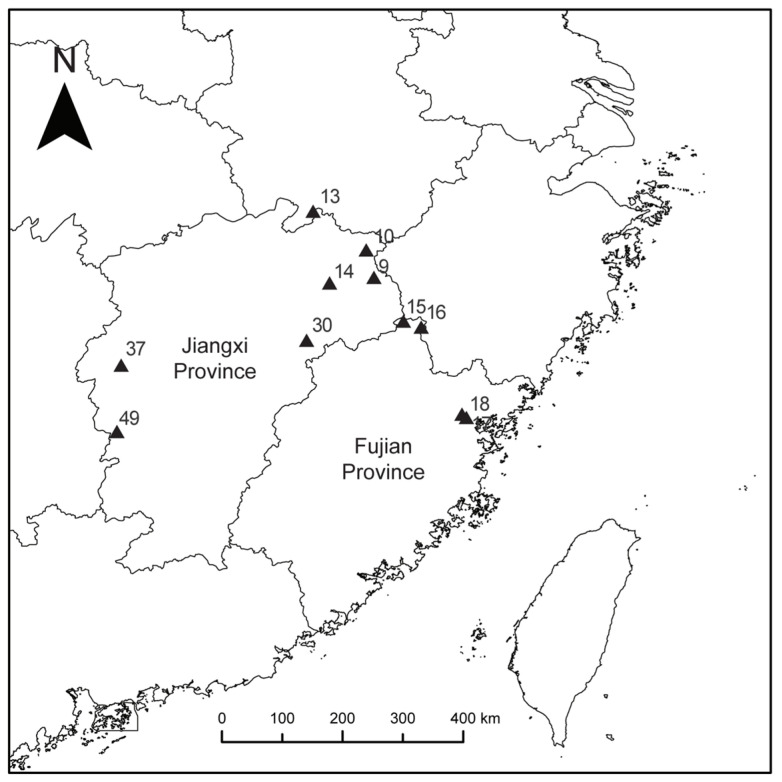
Geographic distribution map of 11 populations of *R. chingii* from Jiangxi and Fujian provinces of China.

The *R. chingii* germplasms used in this investigation were sourced from 11 field areas in the provinces of Jiangxi and Fujian (Supplementary Table S9). The following figure displays the collection’s geographic spread (Figure 6).

### 5.2. DNA Extraction and Library Construction

In summer, healthy leaves were gathered, instantly frozen in liquid nitrogen and then moved to a refrigerator at −80 °C. Following the manufacturer’s instructions, high-quality genomic DNA was isolated from recently frozen *R. chingii* leaf tissue using the Plant Genomic DNA Kit (Magen, Guangzhou, China). Each DNA sample was evaluated for purity and concentration in order to guarantee the caliber of the created library. Following successful completion of the genomic DNA test, library building followed the Hyper-seq protocol to the letter [44]. High-throughput sequencing was carried out using the Illumina NovaSeq 6000 platform (Illumina, San Diego, CA, USA). Fastp (version: 0.20.1; Parameter: Default parameter) was used for filtering and quality assurance [84]. BWA (version: 0.7.17; parameter: mem) comparison analysis was performed for each sample [85], and the filtered clean reads were compared to the reference genome.

### 5.3. Identification of SNPs and InDels

Based on the comparison of the result files, GATK (version: 4.2.5.0; parameter: variant filtration) [86] was used in order to reduce the proportion of false positives and obtain high-quality SNPs and InDels. The identified SNPs and InDels were filtered separately according to the hard-filtering standard recommended by GATK officials. The specific filtering standards applied are as follows:

Criteria for SNPs are as follows: “QD < 2.0 || QUAL < 30.0 || SOR > 3.0 || FS > 60.0 || MQ < 40.0 || MQRankSum < −12.5 || ReadPosRankSum < −8.0”;

Criteria for InDels are as follows: “QD < 2.0 || QUAL < 30.0 || FS > 200.0 || MQ < 40.0 || ReadPosRankSum < −20.0”.

The SNPs and InDels identified were annotated using ANNOVAR [87] to identify the genes destroyed by the mutation to assess the impact of the mutation on the body.

### 5.4. Genetic Diversity

Population genetic parameters and population index (Fst) values were calculated with Stacks software (version 2.65). Molecular AMOVA was completed using the poppr.amova function analysis in Rstudio. The 10 field populations of *R. chingii* from different regions of China were subjected to this method, except population 18, which only has one accession.

### 5.5. Population Structure

Based on GATK hard-filtering the remaining mutation result file, vcftools (version 0.1.16; parameters: -MAF, -max-missing, min-alleles, max-alleles, remove-indels) [88] was used to eliminate MAFs (minor allele frequencies) lower than 0.05 and genotype deletion ratios greater than 20%, and only second-order SNP mutation sites were retained. Finally, the remaining variation sites, after filtering, were used for population structure analysis.

A phylogenetic tree is a branch diagram that describes the order of differentiation between populations and is used to represent the evolutionary relationship between populations. According to the similarities and differences in physical or genetic characteristics of the population, we can infer how closely related they are. Using the neighbor-joining method in PHYLIP (version 3.696; parameter: neighbor), the evolutionary tree (NJ tree) was constructed. Subsequently, ggtree, an R package, was used for visualization based on the tree file (Newick format).

PCA is a method of statistical analysis and simplification of data sets. In genetics, it is mainly used in cluster analysis, which clusters individuals into different subgroups according to the principal component based on the degree of variation difference between the samples of a population. GCTA (version: 1.93.2; parameters: -GRM, -PCA) [89] was used for the PCA analysis.

ADMIXTURE software was used to estimate the maximum likelihood of individual ancestors from multi-site SNPs genotype data sets and to estimate the optimal number of ancestors; that is, the population was divided into several subgroups, where the number of subgroups was called K. Normally, a range of K from 2 to n can be preset, since it is not known how many subpopulations this population actually contained. Software simulation in the case of K = x was carried out by calculating how groups were based on a Bayesian algorithm and the origin of each individual for each composition. For the simulation results of each K value, the software calculated a CV error value and maximum likelihood value, and the best K value could be selected according to both the error value and maximum likelihood value.

ADMIXTURE (version: 1.3.0; parameters: -cv inputFile K) [90] was used for population genetic structural analysis, with K values ranging from 2 to 10.

### 5.6. Core Germplasm Screening and Evaluation

The goal of the core germplasm is to use the fewest genetic resources possible while optimizing the genetic variety of the whole resource population, taking into account geographic distribution. The process of removing the core germplasm from all samples of currently available genetic resources using certain techniques is known as “core germplasm construction”. Originally, core sets were created using phenotypic data that included morphological and agronomic traits. However, currently, molecular markers are the principal method to objectively measure genetic diversity. In order to assess the accuracy of germplasm screening, principal component analysis was performed on both the original and screened core germplasm samples in this study. Genocore was utilized for the screening process [48]. 

## Figures and Tables

**Table 1:** Genetic diversity within and among 132 *R. chingii* accession genotypes.

Pop ID	10	13	14	15	16	17	18	30	37	49	9
Polymorphic Sites	142,311	142,562	346,555	57,793	231,860	45,178	26,621	43,356	92,739	70,498	184,274
%Polymorphic Loci	17.5723	17.6693	33.7596	10.0109	25.0310	8.4686	7.3391	8.6978	13.0160	10.7878	21.0269
Num Indv *	4.4886	4.8872	10.8207	1.7818	7.1989	2.0501	1	2.3320	3.8976	2.7428	5.2249
MAF **	0.9541	0.9543	0.9319	0.9637	0.9425	0.9698	0.9633	0.9721	0.9643	0.9667	0.9459
Observed heterozygosity	0.0413	0.0429	0.0406	0.0460	0.0407	0.0459	0.0734	0.0309	0.0326	0.0340	0.0505
Observed homozygosity	0.9587	0.9571	0.9594	0.9540	0.9593	0.9541	0.9266	0.9691	0.9674	0.9660	0.9495
Expected heterozygosity	0.0586	0.0580	0.0900	0.0424	0.0747	0.0352	0.0367	0.0340	0.0450	0.0410	0.0686
Expected homozygosity	0.9414	0.9420	0.9100	0.9576	0.9253	0.9648	0.9633	0.9660	0.9551	0.9591	0.9314
Pi ***	0.0753	0.0751	0.1078	0.0627	0.0915	0.0532	0.0734	0.0466	0.0580	0.0549	0.0897
Fis ****	0.0714	0.0686	0.1742	0.0269	0.1186	0.0119	0	0.0276	0.0513	0.0378	0.0828

* Num Indv: number of individuals per locus values; ** MAF: main allele frequency; *** Pi: nucleotide diversity; **** Fis: fixation index.

**Table 2:** Analysis of molecular variance (AMOVA) results for the 10 field populations of *R.chingii* from different regions of China *.

Source of Variations	Df **	Sum of Squares	Covariance Components	Percentage of Covariance (%)	Phi (*p* < 0.05)
Between populations	9	35,769.45	286.0839	37.4036	
Within samples	121	57,931.61	478.7737	62.5965	0.3740
Total	130	93,701.07	764.8575	100	

* Since there was only one material in Population 18, which did not meet the prerequisites of the AMOVA, the data in the table do not include 18-1. ** Df: degrees of freedom.

**Table 3:** Correlation index of genetic diversity between core and noncore germplasms * under different sampling proportions.

Sample Population	Sampling Proportion	Sample Numbers	Polymorphic Loci Numbers %	Observed Heterozygosity	Observed Homozygosity	Observed Alleles Number (Na)	Effective Allele Number (Ne)	Shannon’s Information	Nei’s Gene Diversity
Core germplasm	25%	19	86.99	0.1791	0.8209	1.9489	1.4256	0.6027	0.6078
Noncore germplasm	25%	13	80.75	0.1874	0.8162	1.9121	1.4208	0.5908	0.5873
Core germplasm	50%	38	91.50	0.1833	0.8167	1.9963	1.4293	0.6192	0.6341
Noncore germplasm	50%	27	87.39	0.1908	0.8092	1.9816	1.4264	0.6095	0.6250
Core germplasm	100%	77	95.65	0.1868	0.8132	2.0000	1.4360	0.6299	0.6383
Noncore germplasm	100%	55	92.39	0.1892	0.8108	1.9998	1.4290	0.6199	0.6359

* Genocore determined that there are 77 strains of core germplasms, 100% of genotype coverage, and 55 strains of noncore germplasms remaining.

## Data Availability

Data sharing is available upon request.

## Supplementary Materials

The following supporting information can be downloaded at https://www.mdpi.com/article/10.3390/plants13050618/s1, Table S1: Statistics of data quality control results. Table S2: Mean sequencing depth and coverage of individuals. Table S3: Number and frequency of alleles within *R. chingii* wild populations. Table S4: SNPs and InDels count, distribution frequency and mean depth of each chromosome. Table S5: Specific variation statistics for each sample. Table S6: The specific number and proportion of SNPs and InDels annotations. Table S7: Value of the fixation index (Fst). Table S8: Coreset coverage. Table S9: population information. Table S10: Basic Population Genetic Parameters. Figure S1: SNP Density Plot across the Seven Chromosomes of *R. chingii* (LG01-07) Representing Number of SNPs within 1 Mb Window Size. Figure S2: INDEL Density Plot across the Seven Chromosomes of *R. chingii* (LG01-07) Representing Number of INDELs within 1 Mb Window Size. Figure S3: Deletion Density Plot across the Seven Chromosomes of *R. chingii* (LG01-07) Representing the Number of Deletions within 1 Mb Window Size. Figure S4: Line chart of cross-validation error.

## References

[1] Yin Y., Jing Z., Zhang K., Liu X., Li S., Liu H. (2019). Study on Ecological Suitability Regionalization of *Rubus chingii*. Mod. Chin. Med..

[2] Editorial Committee of Flora of China, Chinese Academy of Sciences (1990). Flora of China.

[3] Jin L., Li C., Zhan S., Li X., Hua J. (2022). Chromosome count and estimation of genome size of *Rubus chingii* Hu. Mol. Plant Breed..

[4] Guan Y., Qu B., Wang H., Chen L., Li H., Guo X., Liu J., Liu H., Zhang R. (2023). Research progress of Raspberry and its mature fruit. Chin. Arch. Tradit. Chin. Med..

[5] Sheng J.Y., Wang S.Q., Liu K.H., Zhu B., Zhang Q.Y., Qin L.P., Wu J.J. (2020). *Rubus chingii* Hu: An overview of botany, traditional uses, phytochemistry, and pharmacology. Chin. J. Nat. Med..

[6] He B., Dai L., Jin L., Liu Y., Li X., Luo M., Wang Z., Kai G. (2022). Bioactive components, pharmacological effects, and drug development of traditional herbal medicine *Rubus chingii* Hu (Fu-Pen-Zi). Front. Nutr..

[7] Chinese Pharmacopoeia Commission (2020). Pharmacopoeia of People’s Republic of China. Part I.

[8] Yu G., Luo Z., Wang W., Li Y., Zhou Y., Shi Y. (2019). *Rubus chingii* Hu: A Review of the Phytochemistry and Pharmacology. Front. Pharmacol..

[9] Cheng D., Lei Y., Xie J., Su X., Hu Y., Li C. (2012). Research progress on chemical constituents and pharmacological effects of Fupenzi. J. Chin. Med. Mater..

[10] Ke H., Bao T., Chen W. (2019). Polysaccharide from *Rubus chingii* Hu affords protection against palmitic acid-induced lipotoxicity in human hepatocytes. Int. J. Biol. Macromol..

[11] Li H., Li Y., Zhang Y., Tong L., Sa Y., Sun W. (2023). *Rubus chingii* Hu relieved the polycystic ovary syndrome with enhanced insulin sensitivity through inhibiting TXNIP/NLRP3 inflammasome signaling. Gynecol. Endocrinol..

[12] Kong Y., Hu Y., Li J., Cai J., Qiu Y., Dong C. (2022). Anti-inflammatory Effect of a Novel Pectin Polysaccharide from *Rubus chingii* Hu on Colitis Mice. Front. Nutr..

[13] Ping J., Yan C., Zhu Y., Li J., Hu Y. (2022). Nutritional analysis of raspberries from different areas. Xiandai Hortic..

[14] Sun J., Shen X. (2017). Progress in medicine research and fresh fruit industry analysis of *Rubus chingii* Hu. Bull. Sci. Technol..

[15] Chen Z., Jiang J., Shu L., Li X., Huang J., Qian B., Xu H. (2021). Combined transcriptomic and metabolic analyses reveal potential mechanism for fruit development and quality control of Chinese raspberry (*Rubus chingii* Hu). Plant Cell Rep..

[16] Zhu C. (2015). Cultivation technology and exploitation approach of *Rubus chingii* Hu. Modern Agric. Sci. Technol..

[17] Lv W., Rao J., Bian T. (2018). Introduction domestication and propagation of raspberry in east China. Agric. Technol. Equip..

[18] Hu L., Hua J., Ji Q. (2021). Key techniques of standardized production of palmleaf raspberry. Southeast Hortic..

[19] Yao X., Zhu W., Huang H., Zeng Y., Yu W. (2021). Effective medicinal ingredients and screening of excellent germplasm in *Rubus chingii*. China J. Chin. Meteria Medica.

[20] He Q., Liu B.T., Zhou Z.D., Fang R., Yang S.Z. (2021). Diversity of *Rubus chingii* germplasm resources based on twig and leaf phenotypic traits. Acta Agriculturae Zhejiangensis.

[21] You X., Liu H., Yu H., Li X., Zhu H.W.J., Li F. (2020). Selection of excellent individual plants of *Rubus chingii* Hu. South China For. Sci..

[22] Gelaw Y.M., Eleblu J.S.Y., Ofori K., Fenta B.A., Mukankusi C., Emam E.A., Offei S. (2023). High-density DArTSeq SNP markers revealed wide genetic diversity and structured population in common bean (*Phaseolus vulgaris* L.) germplasm in Ethiopia. Mol. Biol. Rep..

[23] Meng Q., Manghwar H., Hu W. (2022). Study on Supergenus *Rubus* L.: Edible, Medicinal, and Phylogenetic Characterization. Plants.

[24] Yang J., Yang X.J., Guo F.R., Wang L.J., Gu C.Y., Wang Q., Wang Y.S. (2020). Study on the selection of suitable explants and dedifferentiation conditions for in vitro culture of *Rubus chingii* Hu. J. Anhui Agric. Univ..

[25] Li Y., Deng J. (2017). Study on cultivation and breeding of *Rubus* corchorifolius in China. Forest By-Product Speciality China.

[26] Li L. (2006). Classification and identification of Chinese bramble (*Rubus* L.). J. Anhui Agric. Sci..

[27] Miao L., Gao L., Xi D., Li X., Zhu Y., Zhu H. (2023). Genetic diversity analysis of flowering Chinese cabbage based on SNP molecular markers. Mol. Plant Breed..

[28] Lv Q. (2018). Identification of traditional She medicine Gegongniugen and its confusable species of genus *Rubus* using ITS2 barcode. Chin. Tradit. Herbal Drugs.

[29] Zheng C., Liu Y., Yuan L., Wu Y., Wang J., Fu Y., Peng X. (2022). Identification of *Rubus chingii* Hu and its related confounders by PCR-RFLP. Chin. J. Modern Appl. Pharm..

[30] Chen Y., Chen Z., Jiang J., Liu H., Tang Y. (2022). RAPD analysis of intraspecial and interspecific genetic diversity in *Rubus chingii* Hu. Hubei Agric. Sci..

[31] Sun J., Ren J., Hua J., Shen X., Wang Z. (2021). Phenotype Characteristics and Genetic Analysis Based on ISSR Makers of *Rubus chingii* in East China. Mod. Chin. Med..

[32] Wang L., Lei T., Han G., Yue J., Zhang X., Yang Q., Ruan H., Gu C., Zhang Q., Qian T. (2021). The chromosome-scale reference genome of *Rubus chingii* Hu provides insight into the biosynthetic pathway of hydrolyzable tannins. Plant J..

[33] Jiang J., Jin L., Wang L., Chen Z., Sun J., Li X. (2023). Excavation and analysis of SSR from transcriptome of *Rubus chingii* Hu. Mol. Plant Breed..

[34] Liu X., Shang K., Wang J. (2020). Status and Development Suggestion for Wild Raspberry Resources in East China. Bot. Res..

[35] Guo F. (2020). Analysis of ITS Sequence Polymorphisms in *Rubus* and Breeding of Superior Lines. Master’s Thesis.

[36] Zhong Y., Wang Y., Sun Z., Niu J., Shi Y., Huang K., Chen J., Chen J., Luan M. (2021). Genetic Diversity of a Natural Population of *Akebia trifoliata* (Thunb.) Koidz and Extraction of a Core Collection Using Simple Sequence Repeat Markers. Front. Genet..

[37] Holbrook C.C., Anderson W.F. (1995). Evaluation of a Core Collection to Identify Resistance to Late Leafspot in Peanut. Crop Sci..

[38] Wang J., Hu J., Huang X., Xu S. (2008). Progress in constructing data and evaluating parameters of representativeness for plant core collection. Seed.

[39] Frankel O.H., Brown A.H.D. Plant genetic resources today: A critical appraisal. Proceedings of the International Conference of Genetics.

[40] Arai-Kichise Y., Shiwa Y., Nagasaki H., Ebana K., Yoshikawa H., Yano M., Wakasa K. (2011). Discovery of genome-wide DNA polymorphisms in a landrace cultivar of Japonica rice by whole-genome sequencing. Plant Cell Physiol.

[41] Bhattramakki D., Dolan M., Hanafey M., Wineland R., Vaske D., Register J.C., Tingey S.V., Rafalski A. (2002). Insertion-deletion polymorphisms in 3’ regions of maize genes occur frequently and can be used as highly informative genetic markers. Plant Mol. Biol..

[42] Salathia N., Lee H.N., Sangster T.A., Morneau K., Landry C.R., Schellenberg K., Behere A.S., Gunderson K.L., Cavalieri D., Jander G. (2007). Indel arrays: An affordable alternative for genotyping. Plant J..

[43] Liu B., Wang Y., Zhai W., Deng J., Wang H., Cui Y., Cheng F., Wang X., Wu J. (2013). Development of InDel markers for *Brassica rapa* based on whole-genome re-sequencing. Theor. Appl. Genet..

[44] Zou M., Xia Z. (2022). Hyper-seq: A novel, effective, and flexible marker-assisted selection and genotyping approach. Innovation.

[45] Wang F., Xia Z., Zou M., Zhao L., Jiang S., Zhou Y., Zhang C., Ma Y., Bao Y., Sun H. (2022). The autotetraploid potato genome provides insights into highly heterozygous species. Plant Biotechnol. J..

[46] Fu Y., Jiang S., Zou M., Xiao J., Yang L., Luo C., Rao P., Wang W., Ou Z., Liu F. (2022). High-quality reference genome sequences of two Cannaceae species provide insights into the evolution of Cannaceae. Front. Plant Sci..

[47] Ding H., Zhou G., Zhao L., Li X., Wang Y., Xia C., Xia Z., Wan Y. (2023). Genome-Wide Association Analysis of Fruit Shape-Related Traits in *Areca catechu*. Int. J. Mol. Sci..

[48] Jeong S., Kim J.Y., Jeong S.C., Kang S.T., Moon J.K., Kim N. (2017). GenoCore: A simple and fast algorithm for core subset selection from large genotype datasets. PLoS ONE.

[49] Huq A., Akter S., Nou I.S., Kim H.T., Jung Y.J., Kang K.K. (2016). Identification of functional SNPs in genes and their effects on plant phenotypes. J. Plant Biotechnol..

[50] Jiang X., Fang Z., Lai J., Wu Q., Wu J., Gong B., Wang Y. (2022). Genetic Diversity and Population Structure of Chinese Chestnut (*Castanea mollissima* Blume) Cultivars Revealed by GBS Resequencing. Plants.

[51] Palasciano M., Zuluaga D.L., Cerbino D., Blanco E., Aufiero G., D’Agostino N., Sonnante G. (2022). Sweet Cherry Diversity and Relationships in Modern and Local Varieties Based on SNP Markers. Plants.

[52] Mudaki P., Wamalwa L.N., Muui C.W., Nzuve F., Muasya R.M., Nguluu S., Kimani W. (2023). Genetic Diversity and Population Structure of Sorghum (*Sorghum bicolor* (L.) Moench) Landraces Using DArTseq-Derived Single-Nucleotide Polymorphism (SNP) Markers. J. Mol. Evol..

[53] Wei L., Miao H., Li C., Duan Y., Niu J., Zhang T., Zhao Q., Zhang H. (2014). Development of SNP and InDel markers via de novo transcriptome assembly in *Sesamum indicum* L. Mol. Breed..

[54] Li Y., Colleoni C., Zhang J., Liang Q., Hu Y., Ruess H., Simon R., Liu Y., Liu H., Yu G. (2018). Genomic Analyses Yield Markers for Identifying Agronomically Important Genes in Potato. Mol. Plant.

[55] Guo C., McDowell I.C., Nodzenski M., Scholtens D.M., Allen A.S., Lowe W.L., Reddy T.E. (2017). Transversions have larger regulatory effects than transitions. BMC Genom..

[56] Li Y., Luo X., Peng X., Jin Y., Tan H., Wu L., Li J., Pei Y., Xu X., Zhang W. (2023). Development of SNP and InDel markers by genome resequencing and transcriptome sequencing in radish (*Raphanus sativus* L.). BMC Genom..

[57] Jain A., Roorkiwal M., Kale S., Garg V., Yadala R., Varshney R.K. (2019). InDel markers: An extended marker resource for molecular breeding in chickpea. Public Libr. Sci..

[58] Yang J., He J., Wang D., Shi E., Yang W., Geng Q., Wang Z. (2016). Progress in research and application of InDel markers. Biodivers. Sci..

[59] Salem M., Vallejo R.L., Leeds T.D., Palti Y., Liu S., Sabbagh A., Rexroad C.E., Yao J. (2012). RNA-Seq identifies SNP markers for growth traits in rainbow trout. Public Libr. Sci..

[60] Sjödin P., Bataillon T., Schierup M.H. (2010). Insertion and deletion processes in recent human history. Public Libr. Sci..

[61] Kim S.J., Park J.S., Shin Y.H., Park Y.D. (2021). Identification and Validation of Genetic Variations in Transgenic Chinese Cabbage Plants (*Brassica rapa* ssp. pekinensis) by Next-Generation Sequencing. Genes.

[62] Salgotra R.K., Chauhan B.S. (2023). Genetic Diversity, Conservation, and Utilization of Plant Genetic Resources. Genes.

[63] Leigh D.M., Hendry A.P., Vázquez-Domínguez E., Friesen V.L. (2019). Estimated six per cent loss of genetic variation in wild populations since the industrial revolution. Evol. Appl..

[64] Teixeira J.C., Huber C.D. (2021). The inflated significance of neutral genetic diversity in conservation genetics. Proc. Natl. Acad. Sci. USA.

[65] Butchart S.H., Walpole M., Collen B., van Strien A., Scharlemann J.P., Almond R.E., Baillie J.E., Bomhard B., Brown C., Bruno J. (2010). Global biodiversity: Indicators of recent declines. Science.

[66] Jiang Q., Xu Q., Pan J., Yao X., Cheng Z. (2022). Impacts of Chronic Habitat Fragmentation on Genetic Diversity of Natural Populations of *Prunus persica* in China. Plants.

[67] Yin Q., Wang Y., Li H., Hao J., Meng J., Lu B. (2023). Genetic diversity of wild *Zanthoxylum armatum* by ddRAD-seq. Mol. Plant Breed..

[68] Ding T. (2023). Genetic Diversity Analysis and Molecular ID Card Construction of Ancient Chestnut Trees and Varieties (Lines) in Yanshan.

[69] Gumede M.T., Gerrano A.S., Amelework A.B., Modi A.T. (2022). Analysis of Genetic Diversity and Population Structure of Cowpea (*Vigna unguiculata* (L.) Walp) Genotypes Using Single Nucleotide Polymorphism Markers. Plants.

[70] Hoban S., Campbell C.D., da Silva J.M., Ekblom R., Funk W.C., Garner B.A., Godoy J.A., Kershaw F., MacDonald A.J., Mergeay J. (2021). Genetic diversity is considered important but interpreted narrowly in country reports to the Convention on Biological Diversity: Current actions and indicators are insufficient. Biol. Conserv..

[71] Ellstrand N.C., Roose M.L. (1987). Patterns of Genotypic Diversity in Clonal Plant Species. Am. J. Bot..

[72] Pluess A.R., Stöcklin J. (2004). Population genetic diversity of the clonal plant Geum reptans (Rosaceae) in the Swiss Alps. Am. J. Bot..

[73] Kamnev A., Antonova O.Y., Dunaeva S., Gavrilenko T.A., Chukhina I.G. (2020). Molecular markers in the genetic diversity studies of representatives of the genus Rubus L. and prospects of their application in breeding. Vavilovskii Zhurnal Genet Sel..

[74] Yang Y., Lyu M., Liu J., Wu J., Wang Q., Xie T., Li H., Chen R., Sun D., Yang Y. (2022). Construction of an SNP fingerprinting database and population genetic analysis of 329 cauliflower cultivars. BMC Plant Biol..

[75] Hamrick J.L., Godt M.J.W., Sherman-Broyles S.L. (1992). Factors influencing levels of genetic diversity in woody plant species. New For..

[76] Sun W.H., Chen D.Q., Carballar-Lejarazu R., Yang Y., Xiang S., Qiu M.Y., Zou S.Q. (2021). Genetic diversity and population structure of *Euscaphis japonica*, a monotypic species. PeerJ.

[77] Yin M. (2022). Evaluation of Genetic Diversity for Germplasm Resources of Betula Alnoides.

[78] Geng Y. (2020). Preliminary Construction and Genetic Diversity Analysis of Core Collection of Astragalus.

[79] Chen X. (2022). Population Differentiation of Galinsoga Quadriradiata and Its Effects on Diffusion Processes.

[80] Chen X. (2021). Genetic Diversity Analysis of Primula sikkimensis in Hengduan Mountains Revealed by RAD-seq.

[81] Jing T., Zhu X., Shi C., Ye L., Wen G., Lai W., Lv Z., Zhang G. (2023). Genetic diversity analysis of *Fraxinus mandshurica* based on dd-RAD simplified genome sequencing. Mol. Plant Breed..

[82] Li M., Zhao Z., Miao X., Zhou J. (2013). Genetic diversity and population structure of Siberian apricot (*Prunus sibirica* L.) in China. Int. J. Mol. Sci..

[83] Roy Choudhury D., Singh N., Singh A.K., Kumar S., Srinivasan K., Tyagi R.K., Ahmad A., Singh N.K., Singh R. (2014). Analysis of genetic diversity and population structure of rice germplasm from north-eastern region of India and development of a core germplasm set. PLoS ONE.

[84] Chen S., Zhou Y., Chen Y., Gu J. (2018). fastp: An ultra-fast all-in-one FASTQ preprocessor. Bioinformatics.

[85] Li H., Durbin R. (2009). Fast and accurate short read alignment with Burrows-Wheeler transform. Bioinformatics.

[86] McKenna A., Hanna M., Banks E., Sivachenko A., Cibulskis K., Kernytsky A., Garimella K., Altshuler D., Gabriel S., Daly M. (2010). The Genome Analysis Toolkit: A MapReduce framework for analyzing next-generation DNA sequencing data. Genome Res..

[87] Wang K., Li M., Hakonarson H. (2010). ANNOVAR: Functional annotation of genetic variants from high-throughput sequencing data. Nucleic Acids Res..

[88] Danecek P., Auton A., Abecasis G., Albers C.A., Banks E., DePristo M.A., Handsaker R.E., Lunter G., Marth G.T., Sherry S.T. (2011). The variant call format and VCFtools. Bioinformatics.

[89] Yang J., Lee S.H., Goddard M.E., Visscher P.M. (2011). GCTA: A tool for genome-wide complex trait analysis. Am. J. Hum. Genet..

[90] Alexander D.H., Novembre J., Lange K. (2009). Fast model-based estimation of ancestry in unrelated individuals. Genome Res..

