# Supplementary material for: Genetic Diversity Analysis and Core Germplasm Construction of Rubus chingii Hu
Source: Plants (Basel). 2024 Feb 23;13(5):618. doi: 10.3390/plants13050618 (PMC10934504; doi:10.3390/plants13050618)

Figure S1

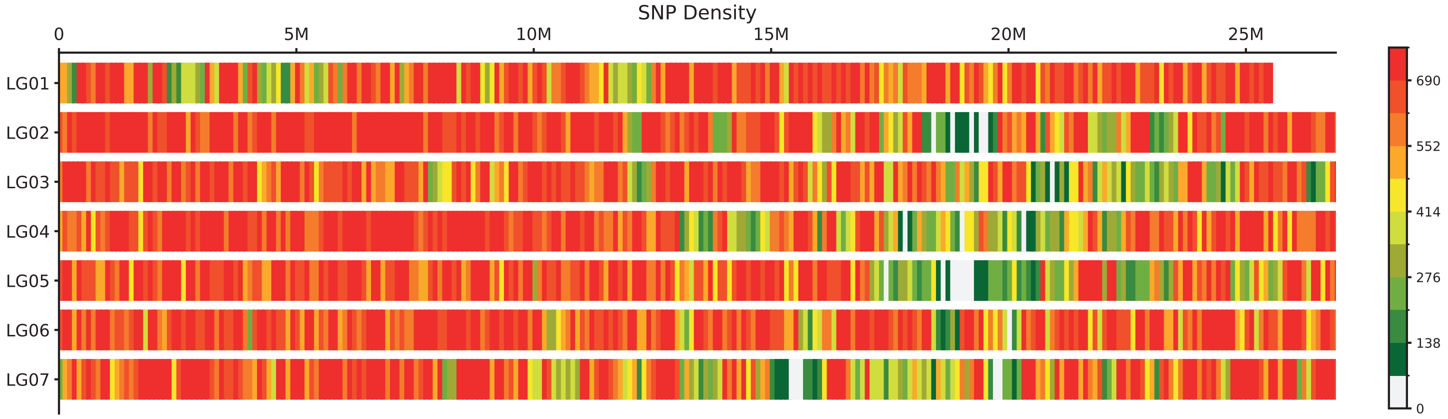

The horizontal axis represents the chromosome length in Mb (M). Different colors correspond to SNP density.

Figure S2

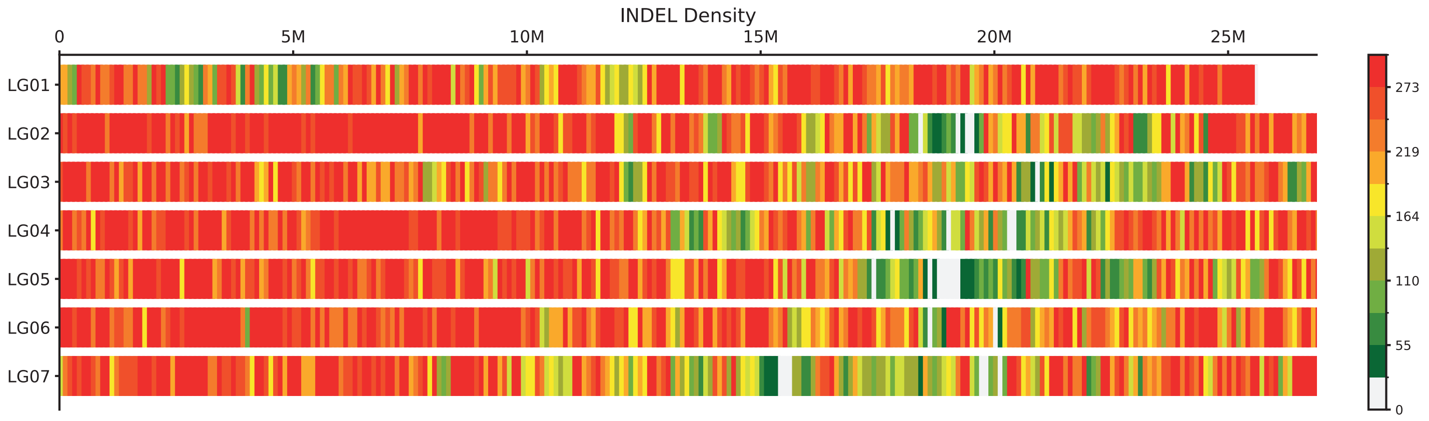

The horizontal axis represents the chromosome length in Mb (M). Different colors correspond to INDEL density.

Figure S3

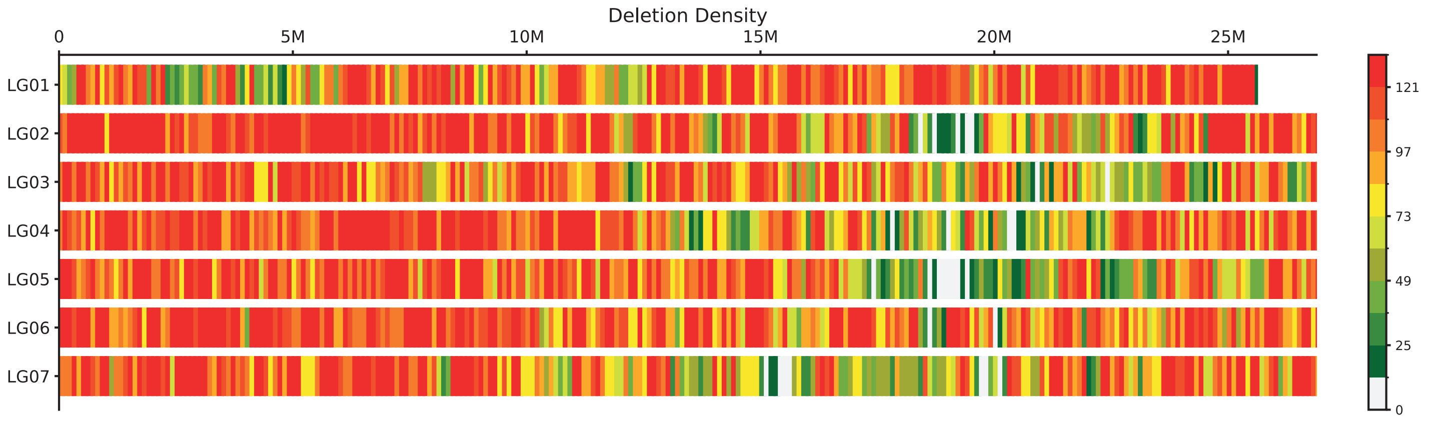

The horizontal axis represents the chromosome length in Mb (M). Different colors correspond to Deletion density.

Figure S4

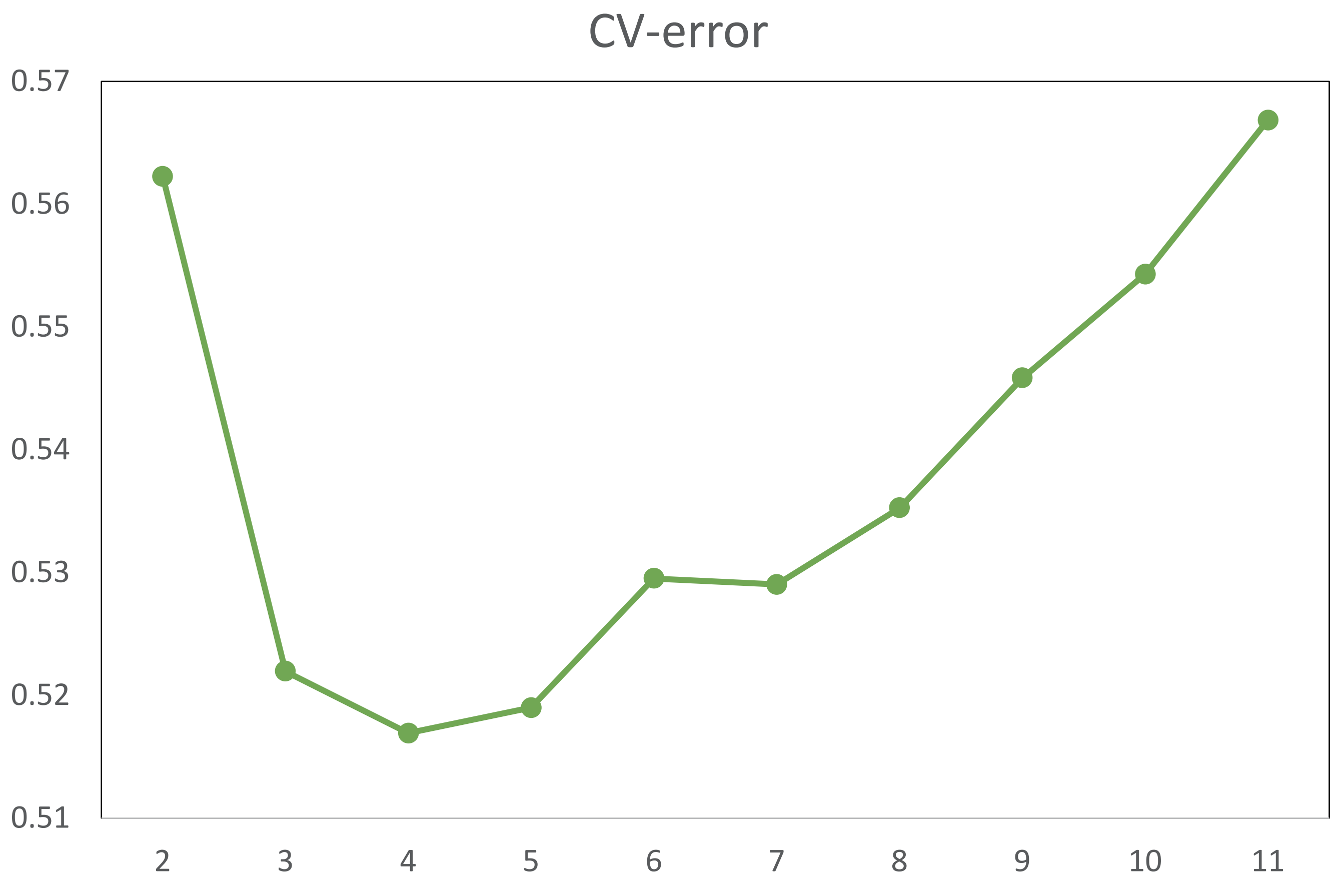

Supplement: Supplementary file 1 [file plants-13-00618-s001.zip › supplementary Figures-all.pdf]
